# Potentially Fatal Ludwig's Angina: A Case Report

**DOI:** 10.7759/cureus.48885

**Published:** 2023-11-16

**Authors:** Prasanna R Sonar, Aarati Panchbhai, Aachal N Lande

**Affiliations:** 1 Oral Medicine and Radiology, Sharad Pawar Dental College and Hospital, Datta Meghe Institute of Higher Education and Research, Wardha, IND; 2 Dentistry, Datta Meghe Institute of Higher Education and Research, Wardha, IND

**Keywords:** tracheostomy, surgical decompression, drainage, incision, ludwig's angina

## Abstract

Ludwig's angina is a condition that could be fatal, causing severe diffuse cellulitis bilaterally that affects the submandibular, sublingual, and submental areas. It has an acute onset and progresses rapidly. A common and potentially deadly complication is airway impairment. Prompt diagnosis and treatment planning have the opportunity to save lives. An elective tracheostomy is recommended for the patient to maintain an open airway, followed by addressing potential affected spaces due to a widespread odontogenic infection. This infection has extended to the neck, causing elevation of the ventral surface of the tongue and floor of the mouth, leading to airway obstruction and the manifestation of stridor. In the latter stages of the illness, additional attention should be paid to maintaining the airway before surgical decompression and antibiotic treatment. In advanced cases, the usual protocol of care still includes surgical drainage of the infection, judicious administration of parenteral antibiotics, and airway management. A case report's objectives are to improve clinical knowledge, facilitate better diagnosis and treatment, and add to the body of medical research by offering a thorough and educational description of a particular patient's experience with this illness.

## Introduction

Ludwig’s angina was coined after Wilhelm Friedrich von Ludwig, who first described this condition in 1836 [1]. Ludwig’s angina is a fatal surgical emergency and is typically a spreading cellulitis of the face, neck spaces, and floor of the mouth with brawny swelling of the submandibular region with elevation and posterior displacement of the tongue, causing airway obstruction [2,3]. The infection is odontogenic in 85% of cases, and other non-odontogenic causes include peritonsillar abscess, parapharyngeal abscess, mandibular fractures, oral piercings or wounds, and submandibular sialadenitis [4,5]. The most common etiology has odontogenic origins. Systemic illnesses such as diabetes mellitus, malnutrition, alcoholism, and acquired immunodeficiency syndrome may be risk factors [6]. Treatment options include high-dose antibiotics, steroids, surgical debridement, and supportive measures. The diagnosis is typically clinical. Before the development of antibiotics, mortality for Ludwig’s angina exceeded 50% [3]. As a result of antibiotic therapy, along with improved imaging modalities and surgical techniques, mortality currently averages approximately 8% [3,4]. Ludwig's angina is an infrequent yet fatal complication. The goal of this case report was to increase awareness and make it easier to find similar instances.

## Case presentation

**Figure 1:**
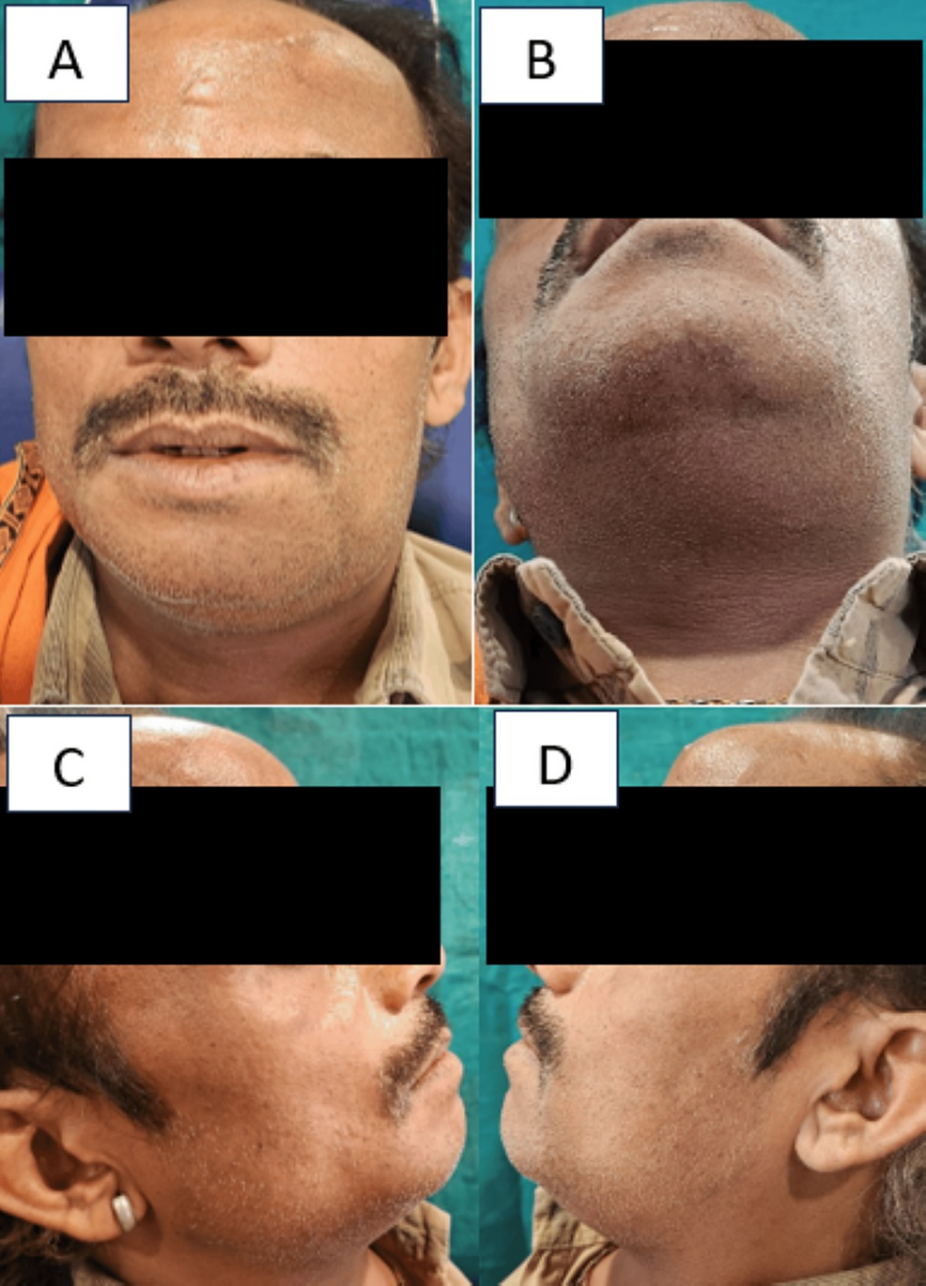
A) Frontal view; B) Image showing submental diffuse cellulitis bilaterally in submandibular, sublingual, and submental areas; C) Right lateral view; D) Left lateral view Image credit: Prasanna R. Sonar

**Figure 2:**
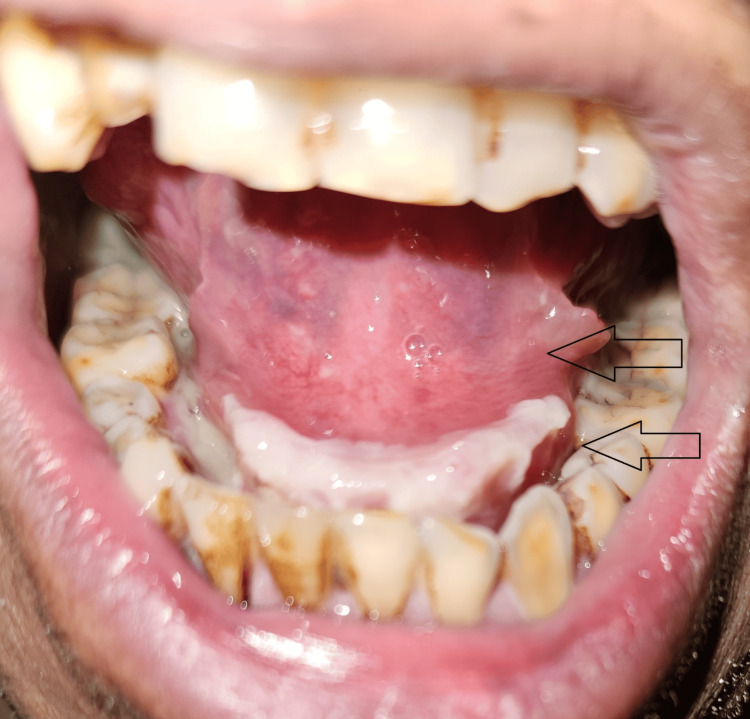
Raised ventral surface of the tongue and floor of the mouth. Image credit: Prasanna R. Sonar

**Figure 3:**
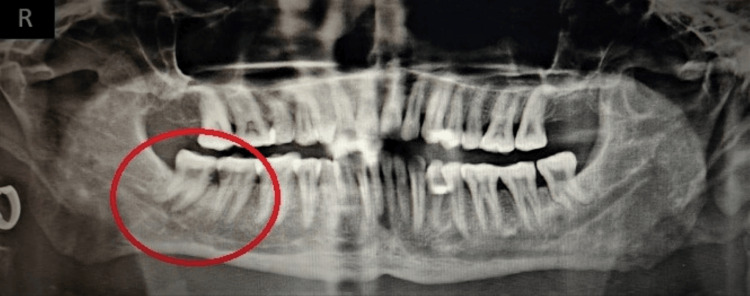
Orthopantomogram showing proximal caries in the 47 and 48 Image credit: Prasanna R. Sonar

A 48-year-old individual arrived at the dental hospital complaining of discomfort and swelling in his lower jaw and neck on the right side, as well as difficulty opening his mouth. The patient had been experiencing these symptoms for a day. There was no significant medical or dental history recorded. Upon physical examination, the patient appeared noxious and had respiratory difficulties. The patient's vital signs showed 146/94 mmHg of blood pressure, 101.8°F temperature, 106 beats per minute of pulse, 18 breaths per minute of respiration, and oxygen saturation in a range of 90 to 94 Sp02. Bilateral submandibular and sublingual gland involvement was present with non-fluctuating extraoral edema, as shown in Figure 1. The mouth opening was inadequate. An intraoral examination identified pus draining from the mandibular right second molar (47) and third molar (48). The raised ventral surface of the tongue and floor of the mouth were inspected, as depicted in Figure 2. The orthopantomogram radiographic evaluation showed proximal caries in the 47 and 48, as depicted in Figure 3.

Ludwig's angina was promptly diagnosed, and the patient was scheduled for surgical decompression under general anesthesia. For the maintenance of airways, however, an elective tracheostomy was planned. Under local anesthesia, the elective tracheostomy was performed, the airway was secured, and general anesthesia was administered. The No. 11 BP blade was used to make separate stab incisions on both the bilateral submandibular and submental regions. By blunt dissection, the bilateral submandibular spaces have been linked to the midline. After the inflammatory exudate was drained, 47 and 48 teeth were extracted. In the context of 47 and 48, an intraoral incision was carried out in the lingual sulcus. Two extraoral incisions were also made in the submandibular region, along with superficial dissection. The pus was removed, and copious amounts of povidone-iodine and saline irrigation were used to flush the wound. The drain was held in place with silk sutures. For five days, the patient was given oral empirical antibiotics in the form of tablets containing 400 mg of metronidazole three times a day and 625 mg of amoxicillin and potassium clavulanate twice a day. Since the culture sensitivity report showed that the patient was sensitive to the empirical antibiotics that were administered, the patient was kept on the same medication for two weeks. The drain was used for postoperative irrigation, and it was taken out after 36 hours. Also, the decayed teeth (47, 48) were extracted. After the surgery, care for the tracheostomy tube was provided. On the fifth day post-operation, once the tube was removed, the skin was securely strapped with a bandage. The patient achieved a good recovery with the disappearance of the symptoms, and the infection was resolved.

## Discussion

Ludwig’s angina is a fatal surgical emergency and is dangerous due to its inherent potential to induce edema, airway distortion, and obstruction [7]. Ludwig's angina is typically caused by dental abscesses and odontogenic infections, such as those involving impacted second and third molars. The primary site of infection for Ludwig's illness, as observed in the case that was presented, is the submandibular region, which is separated into sublingual space superiorly and submaxillary space inferiorly by the mylohyoid muscle. The mylohyoid ridge is penetrated by the infection from the molar in the periapical region, which then proceeds directly to the submandibular, sublingual, and submental spaces as well as indirectly to the pharyngo maxillary and retropharyngeal spaces. This causes a significant, sudden, growing swelling of the neck that surrounds the airway. The mandible, hyoid, and deep cervical fascia limit the infection and edema, which cause the tongue and floor of the mouth to become elevated and posteriorly displaced. This compromise in the airway causes suffocation and a fatal consequence [5,8-11]. 

Patients might have a recent dental pain history. The symptoms that are most frequently mentioned include weakness, chills, fever, and exhaustion. Another common complaint that suggests a more advanced stage of the disease is trismus, which is caused by the infection spreading to the parapharyngeal space. Indications of respiratory involvement include drooling, dysphagia, and tripod posture. Other indications or symptoms include a stiff neck, drooling, tongue swelling, oral pain, and a hoarse voice. The clinical feature of the illness is sometimes referred to as a "bull neck," with increased submental fullness and a receding of the mandibular angle. Patients typically present with submental and submandibular swelling and soreness, along with a fever upon examination. Oral symptoms of infection include swelling of the floor of the mouth, tongue elevation, and soreness in the affected teeth. Although the patient usually does not have lymphadenopathy, common extraoral signs include induration of the submental neck and edema in the upper neck region [9]. The raised ventral surface of the tongue and floor of the mouth were also seen in our case. This is the most significant indicator of Ludwig’s angina.

It is believed that risk factors for Ludwig's angina include advanced age, immunocompromised health, diabetes mellitus, and drinking. These characteristics also raise the chance of complications and death. *Methicillin-resistant Staphylococcus aureus* (MRSA) infections are also more common in people with diabetes, hemodialysis patients, and prolonged hospital stays [11-13]. Age, sex, diabetes, anterior visceral space involvement, kind and side of submandibular involvement, type of infection, and symptoms like trismus and fever are the parameters utilized to examine the risk factors leading to life-threatening consequences. The degree of edema in soft tissues and air accommodation in the tissues, particularly in situations of anaerobic infection, can often be seen on radiographs of the neck and chest. A diagnostic indicator of the intrathoracic extension of the infectious process is the presence of air in the mediastinum or neck [14]. Ultrasonography could be useful in the early detection of cellulitis and abscesses [6,15]. Furthermore, magnetic resonance imaging (MRI) and computed tomography (CT) are used to find mediastinal fluid collections and identify cases of airway edema. Pus collections in the mediastinum and deep neck can be accurately assessed by a CT scan. CT is the preferred imaging modality due to its quicker imaging process. In contrast, while MRI yields images with higher resolution, it necessitates a longer duration for the imaging procedure [13].

Airway maintenance, which requires an assessment of the airway's patency, is the cornerstone of the treatment plan. Broad-spectrum antibiotics are given once infection foci-mandibular molar, pus culture, and sensitivity test have been assessed, incised, and removed with drainage. When an illness has a dental origin that can be found early on, the affected tooth or teeth must be extracted to eradicate the infection's source [16]. Research indicates that the improper use of medications such as nonsteroidal anti-inflammatory medicines, steroids, and antibiotics may impact the disease's course and clinical signs and symptoms, which could delay the accurate diagnosis of the ailment [17]. Even though steroids were used, 71% of the patients in the study underwent surgical decompression and drain placement, indicating that steroids are not the mainstay of care. Nevertheless, the question of whether steroid use warrants surgery is still up for debate [18]. It has been observed that corticosteroids reduce airway and face edema and help antibiotics penetrate the skin. Early surgical surgery improves airway conditions despite conflicting results [13]. It is advised to administer intravenous penicillin G, clindamycin, or metronidazole as antibiotics before receiving the findings of the culture and antibiogram. For the anaerobic cover, metronidazole is advised due to the rising incidence of Bacteroides strains resistant to penicillin [18]. Since over 30% of MRSA and *streptococcal* species are resistant to clindamycin, using clindamycin alone is not recommended [13].

Decompressing the sublingual, submental, and submandibular regions with an external incision and drainage is the standard surgical procedure. A definitive airway is required in cases of significant airway compromise. Depending on the clinical situation, this can be achieved through tracheostomy or endotracheal intubation. Mask breathing will become difficult due to swelling in the neck; therefore, preoxygenating these individuals using any appropriate technique is crucial. Blind oral or nasotracheal intubation is not advised because it might cause severe laryngospasm, worsened edema, and damage to the airways. Furthermore, since supraglottic airway devices cannot be properly positioned as edema advances, their use should be discouraged. A flexible intubating endoscope and an awake intubation technique should ideally be used to treat nasotracheal intubation in seated patients, with the expectation that a surgical airway may be required [13]. In late-stage patients, however, immediate cricothyroidotomy or tracheostomy is recommended [17]. The neck and airway findings of each patient determine the best course of treatment for Ludwig's angina. Tracheostomy under local anesthesia is typically regarded as the best option for treating severe neck infections. Of the 5855 patients with Ludwig's angina who visited hospitals for emergencies in the United States between 2006 and 2014, 47% needed surgical debridement, according to a retrospective analysis [19,20]. In the current case study, the patient needed a mandibular molar extraction along with surgical debridement, antibiotics, and a tracheostomy to prevent airway blockage.

Ludwig's angina is mostly treated with surgical decompression, wide-spectrum antibiotics, and analgesics. The therapeutic strategy is based on the clinical assessment of the patient's airway patency. Ludwig's angina requires a detailed treatment plan and an agreement that can be used as a future reference.

## Conclusions

Ludwig's angina is an unusual, potentially deadly disease. Since early identification and investigation, antibiotic medication, and potentially surgical care are necessary to reduce the associated morbidity and mortality, the dental clinician has to understand how Ludwig's angina presents. The patient's general well-being also depends on thorough, careful observation and supportive care. The presented case emphasizes how important it is for dental professionals to keep an eye out for Ludwig's angina, particularly in patients who have risk factors like tooth decay and other dental infections. To offer these patients complete care, it also highlights the significance of interdisciplinary coordination between physicians, emergency teams, and dentists.
